# Empyema Epidemic

**Published:** 1918-11

**Authors:** 


***Empyema Epidemic.*** By J. L. Miller, and F. B. Lusk, Camp Dodge, Iowa. *Journal A. M. A.*, Aug. 31, 1918.

The authors give an account of a streptococcus pneumonia and empyema epidemic at Camp Dodge in the early part of the present year. Though measles was quite prevalent throughout the preceding winter, pneumonia was very seldom a complication until about May 1st. The ordinary clinical lobar pneumonia, due to the pneumococcus, prevailed until about March 20th; then, abruptly the streptococcus type predominated, with a very great increase in the incidence of the disease. From September 20th until March 20th, the period of ordinary pneumonia, 276 cases were treated; from March 20th to May 10th, 400 patients with pneumonia entered the hospital. The pneumonia during the early autumn was mild, though acute, and the course of the disease was short. In this series of 276 cases, empyema was present in thirty-one, or 11.2 per cent. In these early cases, however, there was a marked tendency to multiple pus foci, and the mortality from empyema was high. Type II, typical and atypical cases, were twice as numerous as those of Type I and Type IV. Those of Type III were only 7.6 per cent. of the whole. The colored troops were much more susceptible than the white, and furnished nearly as many cases, though they were only one sixth of the total strength. Empyema was more fatal among the colored troops though less frequent. The mortality in the thirty-one empyemas, was 61.3 per cent. The epidemic of streptococcus pneumonia appeared suddenly between March 18th and 20th, continued with great severity for six weeks, then gradually became less intense, still continuing, however, until May 10th, at the rate of four or five cases daily. Its virulence, however, was less marked after the first three weeks. It was immediately recognized by the ward surgeon as a different form. "Evidence of severe intoxication appeared very early; empyema became very frequent and developed extremely early, two patients entering the hospital with pleural exudate who had been drilling the day previous. While the involvement in the lung maintained a lobar type, clinical evidence of complete consolidation was far from constant. Dulness with suppressed breathing and subcrepitant râles, but inconstant or localized bronchial breathing, were the usual findings; rusty sputum, provided there was expectoration, was the rule. Early roentgenoscopy showed that the infiltration was lobar in character. The development of an exudate was often exceedingly difficult to determine by the ordinary physical findings, aided by the roentgen ray, and it became necessary to resort to frequent exploratory aspirations. These were often repeated several times before the fluid could be located. Early this exudate was only moderately turbid, contained numerous polynuclear leucocytes and showed on smear short chain streptococci. Gradually the fluid became definitely purulent." The bacteriologic findings in ninety-five of these exudates showed pure streptococci in eighty-eight, all being hemolytic. At the outbreak of the epidemic, a special effort was made to ascertain the predisposing causes or evidence of any infection antedating the onset. Mild sore throat was not uncommon as a preceding symptom, and often in apparent good health it was ushered in with a chill. Some organizations furnished more cases than others. The roentgen ray was found useful as an aid to early diagnosis and localization. The colored soldiers developed empyema in 20 per cent. of the pneumonias, and white soldiers in 45 per cent., and the empyema mortality was much less in the former, while that of streptococcus pneumonia was nearly twice as great. Arthritis as a complication was rare, and so was erysipelas. The treatment is briefly mentioned. All patients were put in special pneumonia wards and isolated by sheet curtains. In the early days of streptococcus pneumonia, immediate drainage was resorted to, but as the mortality was very high, this was discontinued. When the fluid became purulent, rib resection and drainage were employed. In conclusion they say that streptococcus empyemas might be divided into three groups — "(1) those who die early, no matter what form of treatment is instituted, from acute toxemia; (2) those with multiple pus foci, difficult to detect, because of the inability to locate and drain all foci — these all die; and (3) those who usually recover, either from early operation or aspiration followed by operation; here are included those with moderate toxemia and those with localized pus accessible to drainage."

